# Assessment of the Physical Invasiveness of Peroral Endoscopic Myotomy during the Perioperative Period Based on Changes in Energy Metabolism

**DOI:** 10.3390/metabo13090969

**Published:** 2023-08-23

**Authors:** Daisuke Chinda, Tadashi Shimoyama, Sae Fujiwara, Masatoshi Kaizuka, Kohei Yasuda, Kazuki Akitaya, Tetsu Arai, Yohei Sawada, Shiro Hayamizu, Tetsuya Tatsuta, Hidezumi Kikuchi, Miyuki Yanagimachi, Tatsuya Mikami, Hirotake Sakuraba, Shinsaku Fukuda

**Affiliations:** 1 Division of Endoscopy, Hirosaki University Hospital, Hirosaki 036-8563, Japan; 2 Aomori General Health Examination Center, Aomori 030-0962, Japan; tsimo@hirosaki-u.ac.jp; 3 Department of Gastroenterology and Hematology, Hirosaki University Graduate School of Medicine, Hirosaki 036-8562, Japan; fsae@aomorih.johas.go.jp (S.F.); m.kaizuka@hirosaki-u.ac.jp (M.K.); yasuda-kohei@aomori-city-hospital.jp (K.Y.); k_akitaya@hospital-mutsu.or.jp (K.A.); t_arai@hospital-mutsu.or.jp (T.A.); sawada-y@hirosaki-u.ac.jp (Y.S.); hayamizu@hirosaki-u.ac.jp (S.H.); tatsuta.t@hirosaki-u.ac.jp (T.T.); hidezumi@hirosaki-u.ac.jp (H.K.); hirotake@hirosaki-u.ac.jp (H.S.); sfukuda@hirosaki-u.ac.jp (S.F.); 4 Department of Endocrinology and Metabolism, Hirosaki University Graduate School of Medicine, Hirosaki 036-8562, Japan; yanagi@hirosaki-u.ac.jp; 5 Center of Healthy Aging Innovation, Hirosaki University Graduate School of Medicine, Hirosaki 036-8562, Japan; tmika@hirosaki-u.ac.jp

**Keywords:** esophageal achalasia, esophageal motility disorders, peroral endoscopic myotomy, the physical invasiveness, energy metabolism, indirect calorimeter

## Abstract

A novel treatment method for achalasia of the esophagus and related disorders is known as peroral endoscopic myotomy (POEM). This study aimed to calculate the resting energy expenditure (REE) and evaluated the degree of physical invasiveness based on metabolic changes during the perioperative period of POEM. Fifty-eight patients who underwent POEM were prospectively enrolled; REE, body weight (BW), and basal energy expenditure were measured on the day of POEM, postoperative day 1 (POD 1), and three days after POEM (POD 3). The median REE/BW increased from 19.6 kcal/kg on the day of POEM to 24.5 kcal/kg on POD 1. On POD 3, it remained elevated at 20.9 kcal/kg. The stress factor on POD 1 was 1.20. Among the factors, including the Eckardt score, operation time, and the length of myotomy, the length of myotomy was associated with changes in REE/BW. During the perioperative period of POEM, the level of variation in energy expenditure was lower than that of esophageal cancer surgeries performed under general anesthesia. However, because the length of myotomy is a factor affecting changes in energy expenditure, careful perioperative management is desirable for patients with longer myotomy lengths.

## 1. Introduction

Achalasia is a motility disorder characterized by degeneration of the neural plexus in the muscular layer of the esophagus [1]. This condition leads to the absence of peristaltic contractions in the esophageal body and incomplete relaxation of the lower esophageal sphincter (LES) [2]. As a result, patients experience chronic esophageal symptoms, such as dysphagia, regurgitation, and chest pain [3].

Esophageal achalasia is a rare disease with an incidence of 1.0 per 100,000 individuals [4,5,6]. However, esophageal cancer is often a long-term complication in patients with esophageal achalasia [7]. Although the direct causal relationship between esophageal achalasia and the development of esophageal cancer is unclear, Chino et al. reported that chronic inflammation, mucosal injury, and epithelial hyperplasia resulting from prolonged stasis of saliva and food residues can lead to malignant transformation [8]. Therefore, in addition to the clinical symptoms, consideration of the risk of esophageal cancer development is necessary when determining the treatment options for this rare disease.

Previously, treatment options for achalasia included pharmacotherapy (long-acting nitrates and calcium channel blockers) [1,9], endoscopic botulinum toxin injections [10,11], balloon dilation [12], and surgical therapy (laparoscopic Heller myotomy combined with Dor fundoplication) [13,14]. However, in 2008, a novel treatment method for achalasia, known as peroral endoscopic myotomy (POEM), was first reported by Inoue et al. [15]. This technique involves performing a myotomy equivalent to Heller myotomy through an oral endoscope, offering significant advantages over traditional surgery because of its ability to adjust the length of the muscle incision from the esophagus to the stomach [16]. Currently, indications for POEM include achalasia of the esophagus and related disorders, such as diffuse esophageal spasm [17,18], jackhammer esophagus [19,20], and esophagogastric junction outflow obstruction (EGJOO) [21].

According to clinical practice guidelines published by the Japan Gastroenterological Endoscopy Society [16], POEM is a minimally invasive procedure that leaves no visible scars. However, limited research exists on the degree of physical invasiveness of POEM during the perioperative period, and the extent of such invasiveness remains unclear.

In surgical procedures, stress assessment methods, including evaluations of serum interleukin (IL)-6, serum IL-8, C-reactive protein (CRP), and tumor necrosis factor-α levels, have been investigated [22,23,24,25]. Further, stress evaluation methods have also been reported based on differences in anesthesia dosage during laparoscopic fundoplication, showing variations in catecholamine levels (norepinephrine and epinephrine) [26]. However, currently, there is no established evaluation method for assessing the degree of physical invasiveness.

In pathological conditions, such as trauma or sepsis, which are characterized by pathological stress, inflammatory cytokines are produced, and glucose oxidation is enhanced, leading to increased energy metabolism. Additionally, with greater surgical invasiveness, there is an increase in inflammation and protein catabolism, resulting in an increased endogenous energy supply. This increase in metabolism is believed to be proportional to the invasiveness of the surgical procedure [27,28,29,30]. In our previous studies on the perioperative period of endoscopic submucosal dissection (ESD) for early-stage gastric, colorectal, and esophageal cancers, we used indirect calorimetry to calculate the resting energy expenditure (REE) and evaluated the degree of physical invasiveness based on metabolic changes [31,32,33,34].

Thus, it has become evident that the physical invasiveness of ESD during the perioperative period is milder than that during the surgical procedure [32,33,34]. Therefore, assessing and quantifying fluctuations in energy metabolism during the perioperative period allows for the evaluation of physical invasiveness, and this method has been proven to be useful [31].

In this study, we aimed to investigate the rate of metabolic changes, calculate the stress factor to assess physical invasiveness, and elucidate the factors that influence the degree of physical invasiveness using an indirect calorimeter to measure REE during the perioperative period of POEM for esophageal achalasia.

## 2. Materials and Methods

### 2.1. Patient Characteristics

**Figure 1:**
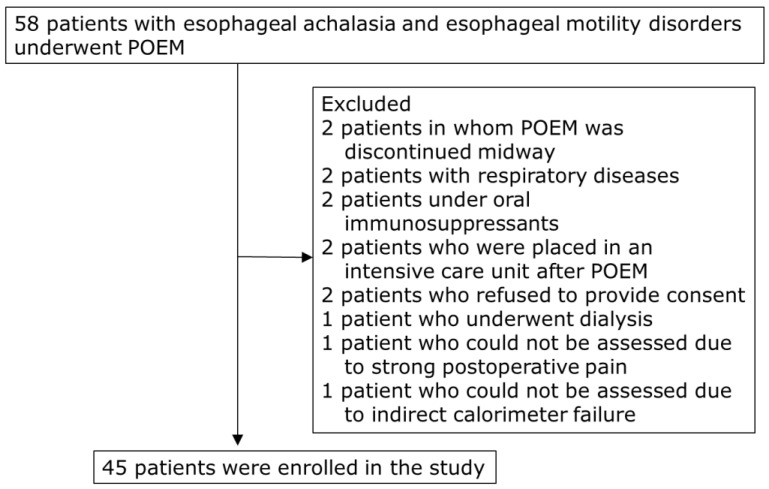
Flow chart of patient selection. POEM, peroral endoscopic myotomy.

This study prospectively enrolled 58 patients with esophageal achalasia and motility disorders who underwent POEM at the Hirosaki University Hospital from July 2017 to March 2023. Patients with respiratory diseases that could affect REE measurements using indirect calorimetry, those undergoing dialysis, and those with other malignant tumors, liver cirrhosis, or thyroid disorders were excluded. Two patients in whom POEM was discontinued midway were excluded (Figure 1). Finally, 45 patients (median age, 57 years; 26 males and 19 females) were included in the study (Table 1).

### 2.2. Ethical Considerations

This study was approved by the Hirosaki University Ethics Committee (approval No. 2013-012, 15 May 2013). The purpose and details of the study were explained to all participants, and written informed consent was obtained.

### 2.3. Definitions/Diagnosis

The diagnosis of achalasia and other esophageal motility disorders was based on a comprehensive evaluation using upper gastrointestinal endoscopy, esophagography, and esophageal manometry. During the endoscopic examination, the presence of vertical surface grooves (pinstripe pattern) or rosette-like folds on the esophageal mucosa and increased resistance at the esophagogastric junction were considered positive findings for achalasia [35,36]. When esophagography revealed the retention of contrast medium in a bird’s beak or “S” shape, it was considered a positive finding for achalasia [3]. Esophageal manometry was performed using high-resolution manometry (HRM) (Starlet; Starmedical Ltd., Tokyo, Japan), and achalasia was diagnosed based on the Chicago classification criteria [37]. On HRM, after catheter insertion, the patient was required to drink 5 mL of water in the supine position for 10 swallows, and the diagnosis was based on the results of this procedure. Type I achalasia was characterized by the absence of panesophageal pressurization after swallowing; type II achalasia was characterized by panesophageal pressurization observed in >20% of swallows; and type III achalasia was characterized by spasms observed in >20% of swallows [37,38]. In addition to the 43 patients with esophageal achalasia, two patients with EGJOO participated in this study. EGJOO is characterized by LES dysfunction and is considered a disorder related to achalasia [21]. Similar to esophageal achalasia, LES insufficiency occurs without the presence of peristaltic disorders in the esophageal body. While there is no definitive treatment for EGJOO, similar to achalasia, in recent years, there have been reports on the effectiveness of POEM for EGJOO, which has been performed in some cases [21].

Symptom severity was assessed using the Eckardt score [39]. To evaluate the preoperative condition of the patient, the total score was calculated by assigning a 3-point score for each of the following symptoms: dysphagia (0 = none, 1 = occasional, 2 = daily, and 3 = with every meal), regurgitation (0 = none, 1 = occasional, 2 = daily, and 3 = with every meal), chest pain (0 = none, 1 = occasional, 2 = daily, and 3 = several times a day), and weight loss (0 = none, 1 ≤ 5% weight loss, 2 = 5–10% weight loss, and 3 ≥ 10% weight loss). Higher scores reflect more severe symptoms (maximum: 12 points), whereas lower scores indicate milder symptoms (minimum: 0).

### 2.4. POEM

All patients underwent POEM performed by two expert endoscopists. As per the standard schedule, the patients were admitted to the hospital the day before the POEM procedure. Patients were kept in a fasting state upon admission, and if there was suspected retention of esophageal contents, upper gastrointestinal endoscopy was performed a day prior using a 3.2 mm channel endoscope (GIF-Q260J; Olympus, Tokyo, Japan), and any contents were removed by suction or net retrieval. POEM was performed under general anesthesia following the surgical technique established by Inoue et al. [15]. The procedure utilized an endoscope (GIF-Q260J or GIF-H290T; Olympus, Tokyo, Japan), needle knife (Triangle Tipknife J; Olympus), waterjet hook knife (KD-620LR; Olympus), and a high-frequency generator with an automatically controlled system (VIO3 or VIO300D; ERBE, Tübingen, Germany).

The specific procedure involved injecting saline solution into the submucosal layer away from the esophagogastric junction, making an incision in the mucosa using a Triangle Tipknife J and creating an entry for the tunnel in the submucosal layer. Subsequently, the tunnel in the submucosal layer was dissected using a knife in spray coagulation mode at 50 W, moving towards the anal side, and a tunnel within the submucosal layer was created up to the gastric cardia. The inner circular muscle of the esophagus was incised from the entry of the tunnel to the gastric side. After successful myotomy, gentamicin 60 mg was sprayed into the incised muscle layer and tunnel, and the entry mucosa was closed using hemostatic clips to complete the procedure.

The day after the POEM procedure, an upper gastrointestinal endoscopy was performed, which confirmed the absence of mucosal necrosis and proper scope passage through the previously constricted esophagogastric junction. The placement of clips at the entry point was also verified, and additional plication with clips was performed, if necessary. Furthermore, after endoscopy, an esophageal contrast study was conducted to ensure that there was no leakage from the esophagus and that the esophagogastric junction was smoothly traversed. After confirming these results, patients were allowed to consume fluids. A liquid diet was initiated three days after POEM, followed by a soft diet on the fourth day. Patients were discharged on the sixth day after the POEM procedure.

One patient (2.2%) required subsequent endoscopic hemostasis, one patient (2.2%) had pneumonia (suspected aspiration pneumonia), and three patients (6.7%) developed a fever (>38 °C). As mentioned in the guidelines [16], frequent pneumoperitoneum during POEM occurs due to the myotomy of the esophagus and stomach muscle layers. Among the 16 patients (31.1%) who experienced pneumoperitoneum in this study, those who had pneumoperitoneum with an impact on hemodynamics were defined as cases in which an abdominal puncture was performed to release the gas.

### 2.5. REE, Basal Energy Expenditure (BEE), and Stress Factor

**Figure 2:**
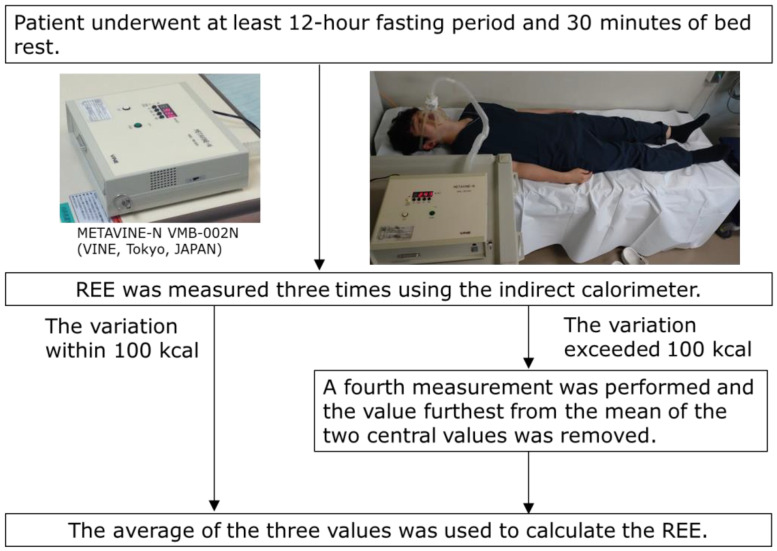
REE measurement profiles of the patients. REE, resting energy expenditure.

REE was measured using an indirect calorimeter (METAVINE-N VMB-002N; VINE, Tokyo, Japan) [31,32,33,34,40]. METAVINE computes the REE using the oxygen concentration and respiration rate in the breath; it does not use carbon dioxide concentration. Each patient fasted for at least 12 h, and REE was measured after 30 min of bed rest in the early morning on the day of POEM, the following day (postoperative day 1; POD 1), and three days after POEM (postoperative day 3; POD 3). Previous studies have demonstrated the accuracy and reproducibility (within 3%) of indirect calorimetry using gas injection tests [41]. In this study, REE was measured three times, and the average value was calculated. If the variation exceeded 100 kcal, a fourth measurement was performed, and the average of the three values, excluding the value furthest from the mean of the two central values, was used to calculate the REE (Figure 2). Additionally, the body weight (BW) of each patient was measured after the expiration test on the day of POEM, POD 1, and POD 3. The REE/BW ratios were calculated using these measurements.

Stress is an indicator of increased metabolic activity [30]. BEE is typically calculated using the Harris–Benedict equation [42], based on Long’s method [43]. REE is defined as the value obtained by multiplying BEE with stress and activity factors. This study focused on a short perioperative period and assumed a constant activity factor. Therefore, the stress factors for POD 1 and POD 3 were calculated by dividing the REE/BEE on the day of POEM by the REE/BEE on POD 1 to determine the activity factor when the stress factor on the day of POEM was 1. Similarly, the REE/BEE on POD 1 was divided by the REE/BEE on POD 3 to determine the activity factor when the stress factor on POD 1 was 1.

### 2.6. Peripheral Leukocytes, Neutrophil Count, and CRP Levels in the Perioperative Period

Blood samples were collected after a 12 h fasting period on the day of POEM, POD 1, and POD 3 in the morning. The number and differential count of white blood cells (WBC) were determined using an XE-5000 (Sysmex, Kobe, Japan) automated hematology analyzer. Serum CRP levels were measured using a JCA-BM6070 analyzer (EOL Ltd., Tokyo, Japan).

Furthermore, Spearman’s rank correlation coefficient was used to investigate the correlation between the rate of change in WBC, neutrophils, and CRP levels from the day of POEM to POD 1, as well as the changes in REE/BW.

### 2.7. Factors Associated with the Rate of Change in REE/BW during the Perioperative Period of POEM

We evaluated the factors that influenced changes in energy metabolism on POD 1, including the Eckardt score, operation time, and the length of myotomy, and patients were divided into two groups based on their median values (the Eckardt score, 5; operation time, 90 min; length of myotomy, 10 cm). The rate of change in REE/BW on POD 1 was compared using univariate analysis. Furthermore, we conducted a multivariate analysis using a generalized linear model to examine the relationship between each factor and the rate of change in REE/BW on POD 1.

### 2.8. Statistical Analysis

Sample size calculation was performed using a two-sided alpha level of 0.05 and a power of 80%. The standard deviation was calculated based on the predicted values derived from previous research data [34] that evaluated the invasiveness of REE during the perioperative period of ESD for gastric cancer. The statistical power of the sample size of 45 was determined to be 0.9676 for REE/BW, and the stress factor was 0.9813.

Statistical analysis of the clinical data was performed using SPSS (version 24.0; SPSS Inc., Chicago, IL, USA) and R (R Foundation for Statistical Computing, version R-3.4.3). Data were presented as medians and interquartile ranges. Statistical differences were analyzed using the Mann–Whitney U and Wilcoxon signed-rank tests. Correlations were assessed using Spearman’s rank correlation coefficients. Additionally, the relationship between each factor and the change in REE was examined using a nonparametric mixed-effects regression model, and a multivariate analysis was conducted using generalized linear models. Statistical significance was set at *p* < 0.05.

## 3. Results

### 3.1. REE, REE/BW, and Stress Factor

Table 2 shows the REE, BW, REE/BW ratio variations, and stress factor during the perioperative period.

**Figure 3:**
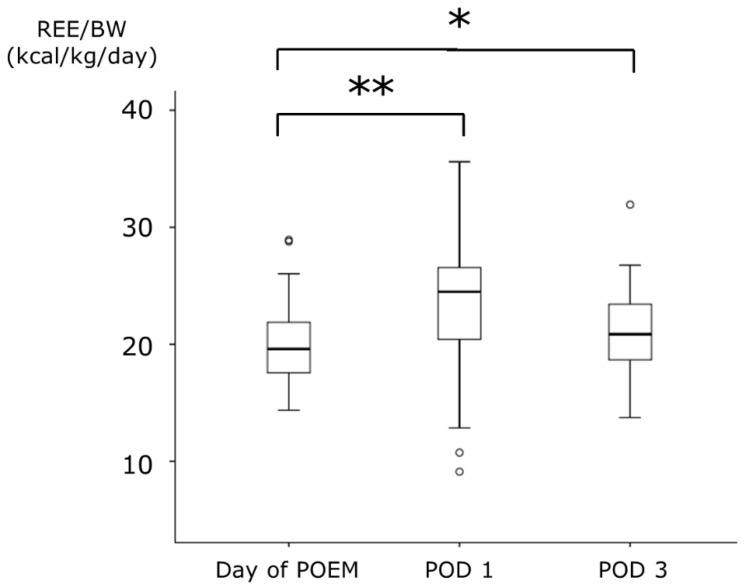
The changes of REE/BW during the perioperative period of POEM. Data are expressed as median (range). * *p* < 0.05, ** *p* < 0.001: compared with the values of Day of POEM. REE, resting energy expenditure; BW, body weight; POEM, peroral endoscopic myotomy; POD, postoperative day.

The median REE on the day of POEM was 1125.0 kcal, which significantly increased to 1389.3 kcal on POD 1. However, on POD 3, the median REE was 1190.3 kcal, with no significant difference compared to that on the day of POEM. There was no significant difference in BW between the day of POEM and POD 1; however, a significant decrease was observed on POD 3. REE/BW increased on POD 1 in 36 of the 45 patients (80.0%). The median REE/BW increased from 19.6 kcal/kg on the day of POEM to 24.5 kcal/kg on POD 1. On POD 3, it remained elevated at 20.9 kcal/kg, which was higher than that observed on the day of POEM (Figure 3).

Regarding the stress factor, when considering the REE/BEE ratio on the day of POEM as 1, it increased significantly to 1.20 on POD 1. However, on POD 3, it decreased to 1.03, and there was no significant difference.

### 3.2. Relationship between Laboratory Findings and Energy Metabolism

Table 3 presents the variations in the WBC, neutrophil, and CRP levels. WBC and neutrophil counts significantly increased on POD 1 compared to the day of POEM (*p* < 0.001), but there was no significant difference between the day of POEM and POD 3. On the other hand, CRP levels increased significantly on POD 1, and although it showed a decreasing trend on POD 3, it remained significantly higher than that on the day of POEM.

Regarding the correlation between the rate of change in REE/BW and the rate of change in WBC, neutrophils, and CRP levels, no significant correlations were observed (r = −0.0321 and *p* = 0.834; r = −0.0661 and *p* = 0.665; and r = 0.2330 and *p* = 0.171, respectively).

### 3.3. Factors Associated with the Change in REE

Table 4 presents the results of the univariate analysis. Regarding the rate of change in REE/BW, significant differences were observed in the length of myotomy. The group with the length of myotomy <10 cm had a significantly lower rate of change compared to the group with the length of myotomy ≥ 10 cm. Meanwhile, no significant differences were observed for the Eckardt score (<5 vs. ≥6) and operation time (<90 min vs. ≥90 min).

Table 5 shows the results of the multivariate analysis using linear models. The factor that influenced REE/BW was the length of myotomy, with an estimated odds ratio of 9.3609 (estimated: 2.2365), indicating a significant difference. However, other factors (the Eckardt score and operation time) did not significantly affect the metabolic changes observed during POEM.

## 4. Discussion

In this study, we investigated the rate of metabolic changes, assessed physical invasiveness, and elucidated the factors influencing the degree of physical invasiveness to measure REE during the perioperative period of POEM for esophageal achalasia. Further, postoperative changes in REE/BW were also examined. Our findings showed a significant increase (25%) in REE/BW on POD 1 compared with that on the day of POEM. The stress factor, defined as REE/BW on the day of POEM, also increased significantly to 1.20 on POD 1. However, on POD 3, there was a trend towards improvement in metabolic changes, with a significant increase of 6.7% in REE/BW compared to the day of POEM, and no significant difference in the stress factor (1.03) was observed.

Surgical invasion induces metabolic changes, and during the perioperative period of surgery, the patient is exposed to various factors such as physical stress from surgery, general anesthesia, and postoperative fasting [28,29,44]. An increase in physical stress increases a patient’s energy requirements [45,46,47]. Furthermore, energy requirements are associated with the degree of physical invasiveness [27]. Therefore, variations in REE measured using an indirect calorimeter can be used to assess physical stress. However, while previous studies have compared REE with BEE to evaluate metabolic changes during the perioperative period [29,48,49,50], only a few studies on REE measurements have been reported. One reason for this is that REE measurements are considered more complex than BEE measurements, and few facilities are equipped with the necessary calorimeters for measurement. Nevertheless, the greatest advantage of this method is that it can be performed using only exhalation, which allows patients to rest in bed, with a measurement time of approximately 5 min per session. Additionally, because BEE is calculated based on height and BW, it does not sensitively reflect small fluctuations in energy metabolism during the short-term perioperative period compared to directly measured REE.

Previous studies have reported an increased REE in the early postoperative period of esophageal surgery under general anesthesia. Sato et al. reported a 31% increase in REE/BW on the first postoperative day compared with preoperative values in Japanese male patients undergoing transthoracic esophageal resection for esophageal cancer [51]. Okamoto et al. found that REE/BW increased to 27.3 ± 3.5 kcal/kg/day on POD 7 compared to 23.3 ± 2.1 kcal/kg/day preoperatively in patients undergoing transthoracic esophageal resection, showing a significant increase [52]. Inoue et al. reported the stress factor as 1.8 on POD 3 in patients undergoing esophageal resection surgery [53]. Based on these results, POEM is considered a less invasive treatment than surgery for esophageal cancer performed under general anesthesia (Table S1). In contrast, Kudo et al. reported a 14.8% increase in the REE on POD 1 after ESD for early-stage esophageal cancer, with a stress factor of 1.11 [32]. This suggests that POEM is a more invasive endoscopic treatment than ESD. One possible reason for the greater invasiveness of POEM compared to ESD is that POEM is performed under general anesthesia, whereas ESD is performed under intravenous sedation. Additionally, while both procedures involve submucosal dissection, POEM involves full-thickness myotomy, whereas ESD involves cutting and removing only the mucosal layer, potentially resulting in less tissue damage and inflammation.

Regarding the inflammatory response, WBC and neutrophil counts significantly increased on POD 1 compared to preoperative values. However, on POD 3, WBC and neutrophil counts improved, and only a mild increase in CRP levels was observed. The number of circulating neutrophils increases in response to physical stress after surgery [54,55,56,57]. This can be attributed to the excessive production of inflammatory cytokines in response to surgical trauma or infection, leading to WBC activation [58]. The results of this study also suggest that the invasiveness of POEM was greatest on POD 1 based on inflammatory markers, with subsequent improvement. Therefore, the REE/BW values and stress factor on POD 1 may reflect the degree of physical invasiveness associated with POEM. However, the lack of correlation between inflammatory markers and the increase in REE suggests that the invasiveness of POEM cannot be solely inferred from the inflammatory reactions observed in blood samples.

In this study, we found that the myotomy length was associated with changes in energy metabolism during the perioperative period of POEM. Previous reports have discussed the prognostic and risk factors for complications of ESD and other surgical procedures [59,60,61]. Age [62,63], body mass index (BMI) [64,65], surgical duration [66], resection area [32], and nutritional status [67,68] are commonly reported factors. Kudo et al. reported that among factors such as age, BMI, total resection area, surgical duration, and sarcopenia, only the total resection area was associated with changes in the REE during the perioperative period of ESD for early-stage esophageal cancer [32]. The authors concluded that one reason for this was the influence of oral commensal bacteria on post-ESD ulcers. Previous studies of gastric ESD have shown that bacteremia is caused by oral commensal bacteria [69]. In POEM, similar to ESD of the esophagus, the invasion of oral commensal bacteria through submucosal tunneling and myotomy during surgery might have contributed to the increase in REE due to early stimulation. However, it is not advisable to shorten the length of the myotomy to minimize its physical invasiveness. It is important to determine the myotomy length based on patient symptoms and improvements in postoperative food intake. Patients with longer myotomies experience greater physical invasiveness and require careful postoperative management.

This study has several limitations. First, it was conducted at a single medical institution. However, in Japan, there are facility standards for POEM, and our institution is certified. Surgery was performed according to the POEM guidelines published by the Japan Gastroenterological Endoscopy Society following standardized procedures. Therefore, similar results can be expected in multicenter studies. Second, it was challenging to establish a control group of healthy volunteers with similar conditions to evaluate the physical invasiveness associated with POEM. Third, we did not measure metabolic changes after POEM beyond the three-day postoperative period. Not measuring metabolic changes beyond POD 3 in the context of POEM could potentially limit our understanding of the procedure’s long-term effects and the overall well-being of patients. If measurements were taken on the seventh day, it might have directly compared with the reported increase in REE/BW for esophageal cancer surgeries, enhancing the differentiation between the physical impact of POEM and esophageal cancer surgeries. However, due to patients being discharged six days post-POEM, we could not conduct these measurements. Furthermore, there is a high likelihood of differing invasiveness between surgeries for achalasia (laparoscopic Heller myotomy combined with Dor fundoplication) and surgeries for esophageal cancer. To better assess the invasiveness gap, endoscopic and surgical approaches for achalasia’s physical impact is essential. However, due to the lack of previous reports on perioperative metabolic variations specific to surgeries for achalasia, we were unable to make such a comparison in this study. Finally, the number of included patients was limited. Our facility is located in Hirosaki City, which has a population of 170,000. Assuming that the incidence rate of esophageal achalasia is 1.0 person per 100,000 individuals, we would expect only 1.7 patients. The population of Aomori Prefecture as a whole, including the medical catchment area, is 1.23 million, making it difficult to include a large number of patients. Consequently, we could not examine numerous factors, and factors such as age, BMI, and nutritional status, which have been reported to be associated with the impact of physical invasiveness in previous studies, were not included as factors influencing POEM. However, these factors were not significant in the univariate analysis in this study.

## 5. Conclusions

During the perioperative period of POEM, the level of variation in energy expenditure is lower than that of esophageal cancer surgeries performed under general anesthesia, which is similar to POEM, suggesting that the physical invasiveness of POEM is relatively low. However, because the length of myotomy is a factor affecting changes in energy expenditure, careful perioperative management is desirable for patients with longer myotomy lengths.

## Figures and Tables

**Table 1:** Characteristics of the patients.

Variables	*n*/Median (Range)
Sex (male:female)	26:19
Age (years)	57 (24–83)
BMI (kg/m^2^)	21.4 (16.2–30.4)
Esophageal motility disorders	
Esophageal achalasia	
Type I	34
Type II	7
Unknown	2
EGJOO	2
Eckardt score	5 (3–10)
Length of myotomy (cm)	10 (5–18)
Operation time (min)	90 (48–158)
Complications	
Bleeding	1 (2.2%)
Pneumonia	1 (2.2%)
Fever (>38 °C)	3 (6.7%)
Pneumoperitoneum	14 (31.1%)

BMI, body mass index; EGJOO, esophagogastric junction outflow obstruction.

**Table 2:** Changes in the REE, REE/BW, and stress factor during the perioperative period of POEM.

	Day of POEM	POD 1	POD 3
REE (kcal)	1125.0 (520.7–1669.0)	1389.3 ** (497.6–2278.0)	1190.3 (510.2–1874.0)
BW (kg)	57.5 (32.7–94.1)	58.7 (33.1–93.0)	56.4 ** (32.2–92.0)
REE/BW	19.6 (14.4–28.9)	24.5 ** (9.1–35.6)	20.9 * (13.7–31.9)
REE/BEE	0.89 (0.59–1.28)	1.06 * (0.47–1.56)	0.94 (0.61–1.36)
Stress factor	1	1.20 * (0.54–1.66)	1.03 (0.74–1.52)

Data are presented as the median (range). * *p* < 0.05, ** *p* < 0.001, vs. POD 1. POEM, peroral endoscopic myotomy; REE, resting energy expenditure; BW, body weight; BEE, basal energy expenditure.

**Table 3:** Changes in WBC, neutrophils, and CRP levels during the perioperative period of POEM.

	Day of POEM	POD 1	POD 3
WBC (/µL)	4900 (1990–11,000)	11410 * (5640–17,150)	5135 (2440–9710)
Neutrophils (/µL)	2681 (919–8389)	9483 * (4698–14,837)	2996 (1093–7260)
CRP (mg/dL)	0.07 (0.00–2.72)	11.41 * (5.64–17.15)	1.71 * (0.39–10.28)

Data are presented as the median (range). * *p* < 0.001, vs. Day of POEM. WBC, white blood cell; CRP, C-reactive protein; POEM, peroral endoscopic myotomy; POD, postoperative day.

**Table 4:** Univariate analysis for the factors associated with changes in the ratio of REE during the perioperative period of POEM.

Variables	n	Day of POEM	POD 1	Changes in the Ratio of REE
Eckardt score				
5	23	19.4	23.7	1.20
		(14.8–28.8)	(10.8–31.4)	(0.60–1.65)
6	22	20.4	24.6	1.20
		(14.4–28.9)	(9.1–35.6)	(0.56–1.57)
Operation time (min)				
<90	22	20.2	24.6	1.17
		(14.8–23.2)	(9.1–31.4)	(1.10–1.66)
≥90	23	19.5	24.3	1.21
		(14.4–28.9)	(15.0–35.6)	(0.84–1.57)
Length of myotomy (cm)				
<10	20	19.5	23.6	1.07
		(14.4–23.2)	(9.1–28.2)	(0.56–1.56)
≥10	25	20	25.6	1.30 *
		(14.7–28.9)	(10.8–35.6)	(0.73–1.66)

Data are presented as the median (range). * *p* < 0.05, vs. myotomy length of myotomy ≥ 10 cm. REE, resting energy expenditure; POEM, peroral endoscopic myotomy; POD, postoperative day.

**Table 5:** Parameter estimates for changes in the ratio of REE/BW using generalized linear models.

Variables	Estimates	SE	Odds Ratio	*p*-Value
Eckardt score	−0.3962	0.7642	0.6729	0.6042
Operation time	−0.2643	0.6844	0.7678	0.6994
Length of myotomy	2.2365	0.7743	9.3608	0.0039

REE, resting energy expenditure; SE: standard error.

## Data Availability

Data presented in this study are available upon request from the corresponding author. The data are not publicly available due to privacy and ethical restrictions.

## Supplementary Materials

The following supporting information can be downloaded at: https://www.mdpi.com/article/10.3390/metabo13090969/s1, Table S1. Comparison of increase rate of REE/BW and stress factor between POEM and esophageal cancer surgeries.
