# Supplementary material for: Assessment of the Physical Invasiveness of Peroral Endoscopic Myotomy during the Perioperative Period Based on Changes in Energy Metabolism
Source: Metabolites. 2023 Aug 23;13(9):969. doi: 10.3390/metabo13090969 (PMC10536107; doi:10.3390/metabo13090969)
Supplement: Supplementary file 1 [file metabolites-13-00969-s001.zip › metabolites-2554769-supplementary.pdf]

Table S1. Comparison of increase rate of REE/BW and stress factor between POEM and esophageal cancer surgeries

| Variables | POEM | esophageal cancer surgeries |
|---|---|---|
| Increase rate of REE/BW (Compared to the day of the operation) | | |
| POD 1 | 25.0% | 31.0% [51] |
| POD 3 | 6.7% | |
| POD 7 | | 17.2% [52] |
| Stress factor | | |
| POD 1 | 1.20 | |
| POD 3 | 1.03 | 1.8 [53] |

REE, resting energy expenditure; BW, body weight; POEM, peroral endoscopic myotomy; POD, postoperative day
